# Municipal bylaw to reduce cosmetic/non-essential pesticide use on household lawns - a policy implementation evaluation

**DOI:** 10.1186/1476-069X-10-74

**Published:** 2011-08-25

**Authors:** Donald C Cole, Loren Vanderlinden, Jessica Leah, Rich Whate, Carol Mee, Monica Bienefeld, Susitha Wanigaratne, Monica Campbell

**Affiliations:** 1 Dalla Lana School of Public Health, University of Toronto, Health Sciences Building, Ste 400, 155 College St. Toronto, ON, M5T 3M7, Canada; 2 Healthy Public Policy, Toronto Public Health, City of Toronto, 277 Victoria Street, 7th Floor, Toronto, Ontario, M5B 1W2, Canada; 3 Public Health Policy and Programs Branch, Ontario Ministry of Health and Long-Term Care, Ministry of Health and Long-Term Care,1075 Bay Street, 11th Floor, Toronto, ON, M5S 2B1, Canada

## Abstract

**Background:**

Pesticide use on urban lawns and gardens contributes to environmental contamination and human exposure. Municipal policies to restrict use and educate households on viable alternatives deserve study. We describe the development and implementation of a cosmetic/non-essential pesticide bylaw by a municipal health department in Toronto, Ontario, Canada and assess changes in resident practices associated with bylaw implementation.

**Methods:**

Implementation indicators built on a logic model and were elaborated through key informant interviews. Bylaw impacts on awareness and practice changes were documented through telephone surveys administered seasonally pre, during and post implementation (2003-2008). Multivariable logistic regression models assessed associations of demographic variables and gardening season with respondent awareness and practices.

**Results:**

Implementation indicators documented multiple municipal health department activities and public involvement in complaints from commencement of the educational phase. During the enforcement phases only 40 warning letters and 7 convictions were needed. The number of lawn care companies increased. Among survey respondents, awareness of the bylaw and the Natural Lawn campaign reached 69% and 76% respectively by 2008. Substantial decreases in the proportion of households applying pesticides (25 to 11%) or hiring lawn care companies for application (15 to 5%) occurred. Parallel absolute increases in use of natural lawn care methods occurred among households themselves (21%) and companies they contracted (7%).

**Conclusions:**

Bylaws or ordinances implemented through education and enforcement are a viable policy option for reducing urban cosmetic pesticide use.

## Background

Growing concern has been expressed by environmental scientists, health researchers, clinicians, and the public about the widespread use of pesticides for lawn and garden applications regarded as "non-essential" i.e. not related to the growing of food, or "cosmetic" i.e. for aesthetic appearances only. These uses contribute to broad exposure to these chemicals as documented by environmental scientists in pesticide run-off in surface waters [1,2] and contamination of groundwater intended for drinking [3]. Human exposure studies have found pesticides among other household exposures [4] and documented pesticide residues associated with pesticide use in homes [5] and on lawns [6]. Potential effects of widespread exposures to pregnant women or children in urban settings are a particular concern [7].

Health researchers have made links between household pesticide use and children's illnesses [7], including cancer [8,9] and developmental impacts [10] from perinatal exposure. Although epidemiological studies are often limited in their ability to interpret cause or ascribe increased risks to specific pesticides, the evidence has been judged to be sufficient to prompt applications of precaution in legislation and regulation to address children's particular vulnerability to pesticides at both national [11,12] and state [13] levels.

Cosmetic pesticide use behaviour is governed by a complex mixture of social and environmental factors, and may be difficult to influence at an individual level [14]. Intensive consumer marketing of the ideal of "the perfect lawn" and of the pesticides needed to achieve it have fostered deeply-entrenched behaviours, neighborhood norms and even municipal ordinances requiring certain standards of lawn and garden care [15]. US surveys have found between one half and three quarters of households use pesticides and/or fertilizers outdoors [16,17] to create and maintain these outdoor spaces according to expectations, while in Canadian surveys prior to bylaw (ordinance) activity, approximately one third to one half of homeowners maintaining lawns and gardens reported using pesticides [18]. Similarly, many lawn care and landscaping companies retained in urban and suburban areas apply pesticides routinely as part of their service packages offered to clients to maintain weed- and insect-free lawns.

In light of the complex array of determinants of householder use of pesticides on lawns, jurisdictional efforts have often begun with programs to reduce the non-essential use of pesticides on public lands (i.e. areas under the direct control of government). For example, between 1995 and 2002 Danish municipalities, with support from the national government, achieved a remarkable reduction of 78% in the tonnes of active ingredients applied in public areas [19]. In a 2001 survey of 448 Ontario municipalities, more than one third did not use pesticides and nearly all had taken steps to substantially reduce or minimize pesticide use on public lands in the previous decade [20]. Approaches taken by nine jurisdictions in the USA, Canada and Europe to reduce residential pesticide use either by households or hired lawn care companies were reviewed by the Canadian Centre for Pollution Prevention in 2004 [21]. Reductions in pesticide use were estimated based on a combination of qualitative and quantitative data, and ranged from marginal (< 10%) to high (> 50%). However, the authors cautioned that "none of the communities had as strong and reliable data as [they] would have liked".

Municipal initiatives to reduce both public and private pesticide use through programs and policies are population-level health interventions that attempt to reduce health risks by changing the social, economic and/or environmental contexts contributing to those risks [22]. Evaluation of municipal initiatives to reduce pesticide use is part of the growing field of population health intervention research in which the ways they bring about change, the value of the interventions, and their effectiveness are all examined.

This paper describes a case where a systematic policy exploration, subsequent bylaw enactment, public education process and enforcement were led by the public health department of a metropolitan North American municipality. In terms of stance, several co-authors have been active participant-observers throughout the process as staff of the health department (more "insiders") while others connected with the university have been involved primarily in evaluation (more "outsiders"). We aimed to document indicators of the policy implementation, which combined both environmental health protection and health promotion components. Our specific research question was "To what extent did resident attitudes and practices change during pesticide bylaw implementation, controlling for demographic and location characteristics?"

## Methods

### Policy & Program Development

#### Setting

Toronto is Canada's largest city and the province of Ontario's capital city. It has a population of approximately 2.5 million people, of which 25% is less than 20 years old, and 20% older than 60 [23]. About half of Toronto's population was born outside of Canada. Just under 50% report a mother tongue other than English or French (the official languages). Income is distributed unevenly, and disparities between rich and poor are growing.

Administratively, the current City of Toronto was created in 1998 by the amalgamation of six former municipalities: the mostly "urban" former city of Toronto; three more "suburban" municipalities (Etobicoke, Scarborough, and North York), and two municipalities with a mix of urban and suburban areas (East York and York) [24]. The only remaining agriculturally zoned areas are in Etobicoke, North York and Scarborough, although small scale growing of fruits and vegetables in home gardens and community garden plots does occur over the summer months in all former municipalities. Visible private and public space is dominated by pavement and grass/lawn coverage on which, historically, the majority of pesticides have been applied.

Public health matters are handled by Toronto Public Health (TPH) under the direction of the Toronto Board of Health (BOH). The BOH is made up of elected officials, citizen and school board representatives, with the Medical Officer of Health (MOH) as the Executive Officer. It sets public health policy and advises City Council, Toronto's main governing and legislative body.

#### Impetus

In Toronto, reductions in pesticide use on public spaces began in the 1980s. Spurred on by concerns of both parents' associations and TPH staff, the Toronto Boards of Education discontinued pesticide spraying on school properties. With the support of environmental organizations (led by the Toronto Environmental Alliance), municipal unions, and staff from multiple City divisions, Toronto City Council adopted the MOH's recommendation in 1998 to restrict pesticide use on all City property. A 97% reduction of herbicide use on general parklands, sports fields and road sides was achieved by 2001 [25].

In 2001, the Supreme Court of Canada upheld the right of a municipality, Hudson Quebec, to restrict pesticide use [26], observing that a 'general enabling clause' in the relevant provincial legislation gave municipalities in Quebec the ability to make bylaws related to health and general welfare. The Ontario *Municipal Act, 2001 *granted municipalities general powers to pass bylaws regulating health and safety concerns when the provincial government does not have legislation governing the activity [27]. Following the Supreme Court of Canada decision, the federal Pest Management Regulatory Agency has also acknowledged the role of municipalities in regulating pesticide use by citizens.

In this permissive federal and provincial context, active municipal councilors championed consideration of a bylaw, the Canadian equivalent to an ordinance. Pressure also came from environmental and health groups, e.g. Toronto Environmental Alliance, the Ontario College of Family Physicians and the Canadian Environmental Law Association. The BOH asked TPH to prepare a document that would generate public discussion on the development and feasibility of various strategies to reduce pesticide use in Toronto and inform policy development.

TPH staff reviewed and synthesized the evidence on potential adverse health effects of lawn and garden pesticides [28]. Based on their findings, the resultant report argued for precautionary actions and for limiting unnecessary uses of commonly used pesticides. In addition, TPH incorporated information from a 2000 telephone survey regarding Toronto residents' awareness about, uses of and attitudes towards lawn pesticides [29] and a 2002 public opinion poll that gauged support for different options to reduce pesticide use [30]. Whether they used pesticides or not, over three quarters of respondents to the poll supported restrictions on pesticides and welcomed information that would help them use safer alternatives.

#### Generation of policy options

TPH incorporated information from an environmental scan of initiatives in other jurisdictions into a policy document [31] with four options: A) public education only, as carried out in Seattle, King County [32]; B) voluntary compliance approach, as in most recycling programs; C) bylaw (all properties), such as Oregon state's Pesticide Tracking Law, also called a "right-to-know" Law [33] and D) bylaw (vulnerable populations only) as in Washington State's children's law around notification of pesticide spraying in schools [34] or New York's Neighbor Notification Law [35].

TPH retained external consultants to undertake a stakeholder consultation on these options in early 2002. They conducted a workshop which brought together 65 stakeholders from pesticide manufacturing companies, lawn care companies, golf course associations, community garden groups, regional conservation authorities, environmental non-governmental organizations, health care provider organizations, school boards, ratepayer groups and governments (provincial Ministry of the Environment, Environment Canada). Workshop results informed six evening public meetings held across the city in the spring of 2002. Approximately 400 people signed in at the meetings and engaged in lively, small group discussions moderated by professional facilitators. A follow-up stakeholder meeting examined the key challenges/barriers the City would face with either a voluntary industry-led initiative or some type of bylaw. Upon consideration of the consultation report, the BOH recommended that Toronto adopt a pesticide bylaw to best protect public health [36].

#### Policy enactment & Program design

In May 2003, Toronto City Council passed a bylaw that "restricts the outdoor use of pesticides on all public and private properties in Toronto." It applied to anyone who might use pesticides outdoors, including homeowners, renters, lawn care companies, golf courses and cemeteries [37]. Pesticides composed of specific low-risk active ingredients such as soaps or oils, biologicals (such as nematodes) or acetic acid, among others, were exempted from the bylaw and had no municipal restrictions on their use (though federal authorities do place some use restrictions). In addition, certain uses of restricted pesticides were permitted under the bylaw: control of pests which infested property or uses related to health protection. Note, the pesticide bylaw did not govern the selling or buying of pesticides, as this falls under provincial jurisdiction. Crop Life Canada, a plant science industry trade association, challenged the City of Toronto's bylaw in court but their case was rejected by successive provincial and federal courts [38,39]

**Figure 1:**
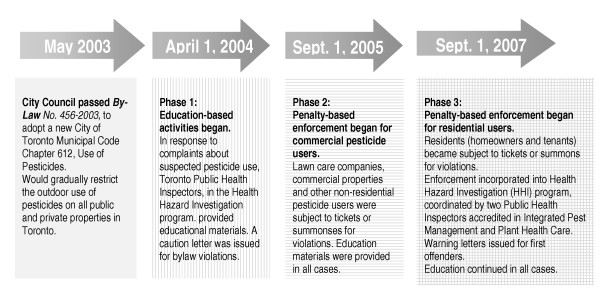
**Toronto Pesticide Bylaw Implementation Phases**.

City Council recognized the need to limit commercial difficulties for lawncare and gardening businesses and to support residents in changing their long-standing methods for lawn and garden care. While a visible enforcement presence in the community was deemed critical to motivate behaviour change among both lawncare professionals and residents, the enforcement strategy was phased-in from 2004 to 2007 (Figure 1), granting time for those accustomed to using pesticides to learn about the restrictions and to adopt alternative methods for lawn and garden care.

A pre-bylaw education program promoting natural lawn and garden care methods such as aerating the lawn, leaving grass clippings on the lawn, spreading organic fertilizers like compost, and applying mulch in garden beds and around trees [40], which can prevent pest problems and minimize the need for pesticides and chemical fertilizers, was substantially expanded. Other City divisions joined and supported the outreach campaigns. For example, Toronto Water contributed funds for advertisements through their drinking water campaign in 2003 and put a reminder in water bills. In keeping with existing evidence on effectiveness of environmental health awareness programs, traditional education/health promotion activities were complemented with more intensive interventions using multiple methods and settings [41]. TPH's education campaign aimed for variety and adaptability, delivering a combination of simple tips and more comprehensive advice, to both residents and commercial users; information through various media, in stores and in several languages and reminders throughout the gardening season to influence key decision points, such as what to do or purchase in spring and fall for a healthy lawn or garden (Table 1).

**Table 1:** Public Education and Outreach Campaign.

Means by which particular audiences were reached with appropriate information:	
**Advertising**	in spring and fall - when people are thinking most about their lawns and gardens - served to remind residents of the bylaw, to balance marketing of traditional pesticides, and to support community acceptance of natural lawn care. In collaboration with Toronto Water and Parks, Forestry and Recreation, 300-500 advertisements were created and placed in major newspapers, community and ethno-cultural newspapers, City guides and newsletters, family and lifestyle magazines, transit shelters and on recycling bins [71].

**City of Toronto website **[72]	had the text of the bylaw, answered frequently-asked questions, included guidance for professional users, provided complaint forms, and made links to relevant information from other City divisions and community organizations. Given the ethnic diversity of Toronto, some material appeared in the City's most commonly spoken non-English languages (French, Spanish, Italian, Portuguese, Russian, Tamil, Chinese and Farsi).

**Toronto Health Connection**	staff responded to public telephone inquiries, processed complaints, sent educational material and provided basic advice on natural lawn care.

**Brochures, fact sheets and lawn signs**	were designed to appeal to residents at all stages of awareness and activity. They contained general information, lawn care and gardening tips, information on how to prevent and deal with specific pest problems; bylaw information; questions to ask a lawn-care company, and information about the lower risk pest control products with no restrictions on use.

**Information in stores**	as both restricted and exempted pesticides remained available for purchase and residents mistakenly assumed that products for sale were "approved" by the City. In consultation with retailers, a "Go Natural" in-store education program was launched in 2005. Go Natural brochures, tear-off sheets, staff aprons, posters and banners were voluntarily posted on store shelves or at cash registers and directed consumers to lower-risk products for certain lawn or garden problems.

**Regular communication**	with professional stakeholders, including landscapers, lawn care companies, arborists and other horticultural professionals to support compliance and their transition to sustainable pesticide reductions.

**Community partnerships**	included 16 environmental and cultural organizations funded to deliver innovative outreach such as workshops, garden tours and radio shows in eight languages. Toronto Public Health also collaborated with academic and community partners to identify communication barriers and explore opportunities to improve multicultural outreach [54].

**Presentations**	by City staff included expert advice through health promotion consultants, Public Health Inspectors, Parks, Forestry and Recreation staff and the Toronto Environmental Volunteers.

**Public events**	included both small community gatherings and large events such as Toronto's Community Environment Days, the Canadian National Exhibition, Canada Blooms, and the Toronto Renovation Forum.

Initial implementation costs projected for the 2004 season [42] were approximately CDN$220K covering seasonal staff time for both bylaw and education work, another CDN$150K for advertising and another CDN$70K for workshops, expert consultation and other expenses (total CDN$450K or approximately US$425K).

### Implementation Indicator Selection

**Figure 2:**
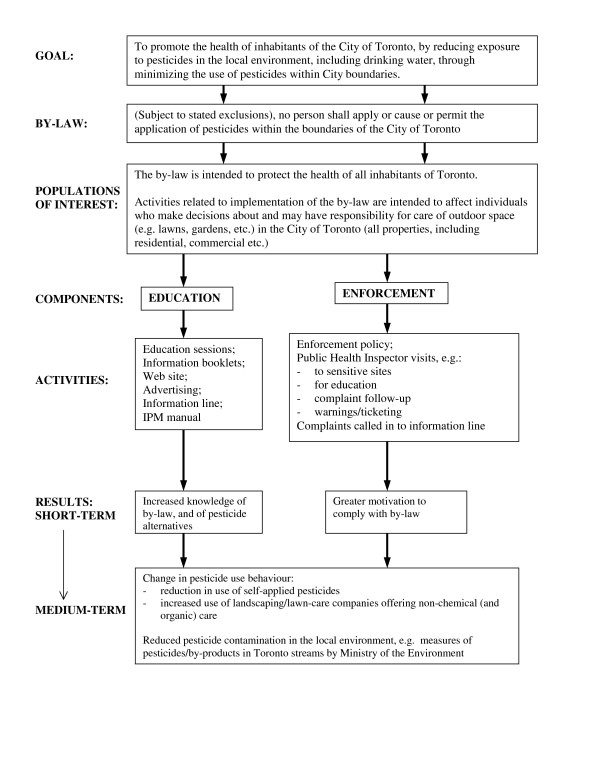
**Logic model for Toronto Pesticide Bylaw**.

To inform monitoring of bylaw implementation, we developed a logic model to lay out in diagrammatic form how the policy was intended to produce results and achieve an overall goal [see Figure 2]. Our logic model identified education and enforcement as two key components, outlined activities for each of these components, and predicted short- and medium-term results.

We also supported a structured literature review to learn from other experiences in documenting household pesticide use practices and evaluating initiatives to reduce such use [14]. Building on this, we interviewed 14 key informants from a wide range of sectors as to potential indicators for evaluating bylaw implementation. The following five potential indicator domains emerged from the literature review and key informants: 1) Enforcement/Legal; 2) Education and Outreach activities, and associated community responses; 3) Economic; 4) Environmental testing; and 5) Medical/Public Health, including urine bio-monitoring and clinical visits.

Data on Enforcement/legal (1) and Education/outreach implementation indicators (2) were most readily available as TPH staff implemented the program. TPH tracks bylaw complaints and the details of complaint investigations through its Toronto Healthy Environments Information System (THEIS) database. TPH also tracked activities relevant to its education and outreach activities (as outlined in Table 1).

The potential economic impacts of a bylaw were an important concern expressed by key informants from landscaping and lawn care companies. Since actual sales data on pesticides or services were not available, we used data on companies in business available from Statistics Canada [(Business Register, Canadian Business Patterns (1998-2005) & (2001-2006)]. These provided a rough indicator of potential economic impacts before and during bylaw implementation (3).

Environment Canada, Ontario Ministry of Environment and Toronto Works and Emergency Services colleagues had done some short-term surface water testing from 1998 to 2000, particularly during high run-off events (4) [43]. Unfortunately, the costs of systematic, regular, long-term surface water monitoring were un-supported at the time the bylaw was implemented. Further, we were unable to implement indicators for domain 5 for several reasons. For bio-monitoring, we were concerned that intra- and inter-individual variation could potentially swamp an exposure reduction effect, the substantial costs involved (minimally an estimated $125K annually over at least five years) were beyond municipal resources, and ethical concerns had been raised during the evaluation of environmental health interventions [44,45]. We explored clinical data systems but those available were incomplete, focused on hospital visits only, and did not adequately identify pesticide exposure in routinely coded data, unlike pesticide exposure incident reporting systems specifically designed for such purposes which have proved very useful in evaluating reductions in other jurisdictions [46].

### Repeat Surveys

#### Design

To track community responses via resident awareness and behaviour over time (domain 2), we turned to the Rapid Risk Factor Surveillance System (RRFSS), a set of ongoing monthly surveys designed to monitor community trends in health risk behaviours among the Ontario population. RRFSS surveys are administered independently by the Institute for Social Research, York University, and consist of questions organized into core and optional modules [47]. TPH led development of optional "pesticides and lawns", "pesticide bylaw" and "pesticide reduction education" modules that were conducted on a monthly basis from 2003 to 2008. The repeated surveys were an appropriate evaluation tool given the phased in approach of pesticide bylaw implementation (See Figure 1). This design approximates the one-group pretest-posttest design most frequently used to evaluate the impact of health programs, though with more 'during' measures, given the phased implementation [48]. Household selection was via random digit dialing procedures. A phone number was called repeatedly until either the survey was completed or the maximum number of 14 calls had been made, at which time the number was considered a 'dead' sample. Within households, the adult with the most recent birthday was selected with no substitutions of more willing household members. Attempts were made to encourage those individuals who initially refused to participate by calling them at least once after they first refused. Consent to participate was verbal, with no address, name or other identifying information collected (i.e. anonymous) as approved by York University's Research Ethics Board, Human Participants Review Subcommittee for generation and use of RRFSS data.

All interviews were completed in English, using Computer-Assisted Telephone Interviewing techniques, which greatly assists in expediting the data editing and cleaning process because logical and quality control checks can be programmed into the questionnaire design [49]. Respondents were asked about either the current (for surveys conducted from April to October) or most recent past (for surveys conducted from October to following spring) gardening seasons. Although this may create some uncertainty in assignation of the relevant gardening season, it was required when surveys were administered in winter months when no gardening was occurring. RRFSS data had indicated that about 50% of households in Toronto had lawns. Because questions about pesticide use were only asked of this subsample of residents, an oversample was implemented in some seasons to ensure a subsample of sufficient size to allow for more detailed analyses of the data.

Socio-demographic variables available included: respondent's gender, pre-tax household income (twelve categories), respondent's highest level of education (four categories from did not graduate from high school to college or university degree) and household location within the City (as defined by the household's municipality prior to amalgamation). Missing data, in addition to item refusals or "don't know", varied from 0 (gender) to 27% (household income). Household size, asked in some years, was on average 2 adults, permitting the estimation of a low income cut-off (LICO) as CDN$27,601 [50]. As this fell towards the upper limit of an income category on the survey ($20,000 to $29,999), all respondents in this category or the lowest (< 20,000) were designated as below the LICO.

For bylaw awareness, all respondents from 2005 on were asked "Some communities have bylaws that limit the outdoor use of pesticides, some are thinking about it and others do not. Do you think that the City of Toronto currently has a bylaw that limits the outdoor use of pesticides?" Further, respondents were asked "Have you seen or heard anything about the Naturally Green/Simple and Effective Lawn Care Campaign in your community? ...includes lawn signs, brochures, and ads in the newspaper which encourage people to avoid pesticides and try pesticide free methods." Responses (Yes/No) became the pesticide bylaw and Natural Lawn Care Campaign awareness dependent variables. Additional questions were asked to understand reasons for reducing pesticide use, using more natural methods and obtaining information on each of these, particularly among the 2005 and 2006 oversamples [51].

For household practices, across all years, respondents were first asked "Does your home have a lawn that you or someone else in your household is taking care of?" If yes, then "Did you or someone else in your household hire or pay a lawn care company to treat your lawn?" and, if yes, then "Did the lawn care company use any pesticides on your lawn to kill weeds or insects?" and "Did they offer to use any natural lawn care/pesticide-free methods such as aeration, over-seeding, hand weeding, or products such as corn gluten?" Similar questions were also asked of respondents with a lawn about applications by "you or someone else in your household" (except for natural lawn care methods in the first year of interest). These became the practices or use dependent variables.

#### Analysis

Data distributions were analyzed with STATA/IC statistical software version 11.0 (2009). Individual level variables (gender, education, pesticide bylaw awareness and Natural Lawn Care Campaign awareness) were weighted to account for the unequal probabilities of selection of one-adult households [52]. Two weights were created for gender and education (gardening 2003-2008), and for pesticide bylaw and Natural Lawn Care Campaign awareness (gardening season 2005-2008), to account for the different time periods in which the questions were asked. Oversamples were given separate values for gardening years, as demographic characteristics of these oversamples varied systematically from regular samples; i.e., more women, greater education, and different location distribution. For key practice variables, confidence intervals were calculated on the first and last year proportions. Bivariate associations were assessed with chi-square statistics.

We then constructed multivariable logistic models with gardening year as the primary independent variable of interest and demographic variables as covariate independent variables (to control for differences across years), with one awareness or use variable as the dependent variable in each model. As associations were observed between awareness variables and between them and gardening year (i.e. increased awareness as bylaw enforcement and education programs rolled out), variable selection was required. The latter examined associations between gender, level of education, income, location, and gardening year as independent variables and respondent awareness or household practice dependent variables. Given the mix of individual and household variables, we conducted sensitivity analyses to examine the effect of using weights that account for individual-level variables versus household variables, and weights that account for different time periods in which the questions were asked. These weights are available from authors upon request. Eventually we settled on application of individual weights as the most inclusive option. Changes in adjusted odds ratios (OR) with 95% confidence intervals were displayed graphically.

## Results

### Implementation indicators

Enforcement/legal - Public Health Inspectors with special training in integrated pest management/plant health care led an enforcement strategy that included proactive visits to schools, golf courses and other properties, participation in educational events in the community and at garden centres, and in-person response to over 3,000 complaints of suspected violations, most occurring early on (see complaint investigations, Table 2). From initiation in 2004 to the first full season of enforcement on lawn care companies and commercial properties (2006), complaints decreased over 80 per cent. This decrease and the low number of convictions required suggest that enforcement and education messages reached much of the professional sector and most came into compliance [53].

**Table 2:** Relevant indicators of Toronto pesticide policy roll-out and program implementation.

Domain	Indicator	Gardening Season				
		
		2004	2005	2006	2007	2008
Enforcement/legal	Bylaw complaint investigations	1672	1118	294	74	127*
	Warning letters issued	NA	6	28	6	0
	Convictions	NA	3	0	1	3

Education & Outreach	Advertisements placed	353	503	335	850**	850
	Website - Pages of content	50	197	230	No info	No info
	Traffic/month	4,754	7,999	12,000		
	General information materials (postcard, brochure, pamphlet)	40,000	20,356	92, 949	89,250	> 75,000
	Technical manual	435	892	482	45	25
	Plastic "pesticide" free lawn signs	3000	1646	300	Discontinued	NA
	Telephone Inquiries	709	588	434	174	318
	Presentations at events	53	74	74	32	20
	Go Natural retail participation	NA	122 stores 83,343 materials	122 stores 98,000 materials	113 stores 53,334 materials	145 30,627 materials
	Fact sheets on natural gardening	NA	NA	> 2500	> 2500	> 2500

*Several lawn care companies introduced a new lower-risk pesticide in 2008, which triggered an increase in complaints, though the pesticide was compliant with the bylaw.

** In 2007 and 2008, TPH initiated an extensive month-long radio ad campaign, which significantly increased the number of ads.

NA = Not applicable

Education/outreach - As can be seen in Table 2, the effort to make residents aware was substantial. In addition to those methods listed, city staff conducted 291 proactive information visits to sensitive sites, such as child care centers and hospitals, and all public and private golf courses and bowling greens. Informal feedback from the community helped identify the need for expert resources on plants and gardens not just lawns. TPH responded by partnering with the Toronto Master Gardeners to produce a series of fact sheets on natural care of flowers and vegetable gardens and promote the information via the internet, during lectures, community events and a telephone information line. Community feedback also resulted in new retail materials, information for the lawn care sector, and particular efforts with ethno-cultural partners [54].

Economic - From 2001 to 2006, the number of landscaping and lawn care sector businesses located in the City of Toronto grew each year, with an overall 30 per cent increase during the period, similar to the increases in companies located anywhere in the Greater Toronto Area (36%) and across Ontario (32%) [53].

### Repeat Survey findings

Response rates across the six years of interest (2003-2008) ranged from 58% (in 2005) to 50% (in 2007). Explicit oversamples of those with lawns occurred for the 2005 (n = 355) and 2006 (n = 179) gardening seasons, resulting in an overall sample of 4,901 respondents.

As can be seen in Table 3, over half of households (55.6%) reported having or caring for a lawn, with greater proportions in the oversampled garden seasons (> 60%) in keeping with intentional selection in the oversamples (e.g. in 2005, 541/1085 or 49.9% in regular sample and 100% in oversample). The proportion hiring lawn care companies remained relatively consistent over time (maximum 26.6% of those with lawns in 2004, minimum 20.3% in 2007, back up to 23.3% in 2008), agreeing with the business data and contrary to the fears of some lawn care spokespeople.

**Table 3:** Respondent* and household^ characteristics, by gardening season (n, %) (Rapid Risk Factor Surveillance Survey, Toronto).

Characteristics	Gardening Season						Totals (N = 4901)
		
	2003 (N = 608)	2004 (N = 609)	2005‡ (N = 1440)	2006‡ (N = 777)	2007 (N = 620)	2008 (N = 847)	
Gender* (weighted counts, wgtd %)	(n = 608)	(n = 607)	(n = 1453)	(n = 795)	(n = 614)	(n = 825)	
Women	316 (52.0%)	353 (58.2%)	795 (54.7%)	459 (57.7%)	344 (56.0%)	457 (55.3%)	2723 (55.6%)
Men	292 (48.0%)	253 (41.8%)	658 (45.3%)	336 (42.3%)	270 (44.0%)	368 (44.7%)	2178 (44.4%)

Education* (wgtd counts, wgtd %)	(n = 608)	(n = 607)	(n = 1453)	(n = 795)	(n = 614)	(n = 825)	
< High school	62 (10.2%)	82 (13.5%)	130 (9.0%)	77 (9.7%)	45 (7.3%)	63 (7.7%)	459 (9.4%)
High school	118 (19.5%)	111 (18.2%)	269 (18.5%)	138 (17.4%)	130 (21.2%)	182 (22.1%)	947 (19.3%)
Some post-2ndy	58 (9.6%)	60 (9.9%)	124 (8.6%)	76 (9.6%)	40 (6.5%)	75 (9.1%)	434 (8.9%)
Completed post-2ndy	361 (59.4%)	347 (57.2%)	904 (62.2%)	496 (62.3%)	394 (64.1%)	494 (59.8%)	2995 (61.1%)
Missing, don't know, refused	9 (1.4%)	7 (1.2%)	26 (1.7%)	8 (1.0%)	5 (0.9%)	11 (1.3%)	66 (1.3%)

**Income^†**							
< low income cutoff	122 (20.1%)	127 (20.9%)	201 (14.0%)	121 (15.6%)	91 (14.7%)	137 (16.2%)	799 (16.3%)
≥ > low income cutoff	357 (58.7%)	344 (56.5%)	842 (58.5%)	433 (55.7%)	336 (54.2%)	460 (54.3%)	2772 (56.6%)
Missing, don't know, refused	129 (21.2%)	138 (22.7%)	397 (27.8%)	223 (28.7%)	193 (31.1%)	250 (29.5%)	1330 (27.1%)

**Municipality^**							
East York	36 (5.9%)	33 (5.4%)	98 (6.8%)	60 (7.7%)	40 (6.5%)	64 (7.6%)	331 (6.8%)
Etobicoke	98 (16.1%)	76 (12.5%)	190 (13.2%)	98 (12.6%)	86 (13.9%)	142 (16.8%)	690 (14.1%)
North York	157 (25.8%)	155 (25.5%)	340 (23.6%)	172 (22.1%)	151 (24.4%)	189 (22.3%)	1164 (23.8%)
Old City of Toronto	168 (27.6%)	183 (30.0%)	388 (26.9%)	215 (27.7%)	174 (28.1%)	241 (28.5%)	1369 (27.9%)
Scarborough	116 (19.1%)	118 (19.4%)	333 (23.1%)	175 (22.5%)	133 (21.5%)	168 (19.8%)	1043 (21.3%)
York	29 (4.8%)	32 (5.3%)	70 (4.9%)	48 (6.2%)	27 (4.4%)	34 (4.0%)	240 (4.9%)
Other	1 (0.2%)	1 (0.2%)	2 (0.1%)	1 (0.1%)	3 (0.5%)	0 (0.0%)	8 (0.2%)
Missing, don't know, refused	3 (0.5%)	11 (1.8%)	19 (1.3%)	8 (1.0%)	6 (1.0%)	9 (1.1%)	56 (1.1%)

**Had a lawn^**							
Yes	333 (54.8%)	289 (47.5%)	896 (62.2%)	479 (61.7%)	308 (49.7%)	421 (49.7%)	2726 (55.6%)
No	265 (43.6%)	312 (51.2%)	529 (36.7%)	294 (37.8%)	308 (49.7%)	421 (49.7%)	2129 (43.4%)
Missing, don't know, refused	10 (1.6%)	8 (1.3%)	15 (1.0%)	4 (0.5%)	4 (0.6%)	4 (0.6%)	46 (0.9%)

**Hired a lawn care company^ **[among those with lawns]							
Yes	81 (24.3%)	77 (26.6%)	193 (21.5%)	97 (20.3%)	72 (23.4%)	98 (23.3%)	618 (22.7%)
No	248 (74.5%)	202 (69.9%)	693 (77.3%)	376 (78.5%)	232 (75.3%)	316 (75.1%)	2067 (75.8%)
Missing, don't know, refused	4 (1.2%)	10 (3.5%)	10 (1.1%)	6 (1.3%)	4 (1.3%)	7 (1.7%)	41 (1.5%)

‡ Explicit oversamples of those with lawns occurred for the 2005 (n = 355) and 2006 (n = 179) gardening seasons

**†**The cut-offs are based on income before taxes for a 2 person household in a community size of 500,00 and over. Since the cut-off for a 2 person household in a community size of 500,000 and

over is 27,601, a value within an income category ($20,000 to $29,999), all respondents within this category were designated below the LICO. All respondents within income categories > 30,000 were designated above the LICO.

Across gardening seasons, the (weighted) proportion of respondents indicating awareness of the Toronto pesticide bylaw increased from 50.6% (2005) to 69.2% (2008) (Table 4). Among those with lawns, reported use of pesticides on their lawn decreased, both by a company they hired (14.7% in 2003 to 4.5% in 2008) or by a household member (24.6% in 2003 to 11.2% in 2008) with no overlap of 95% confidence intervals (Table 5). Respondent awareness of the Natural Lawn Care Campaign among those with lawns showed little change (36.8% in 2005 to 37.8% in 2008), but use of natural lawn care practices by a company they hired (4.8% in 2003 to 11.9% in 2008) or a household member (45.3% in 2004 to 66.3% in 2008) did increase (confidence intervals also non-overlapping).

**Table 4:** Respondent* & household^ lawn care awareness and practices, City of Toronto, 2003-2009 All households (n, %).

Lawn Care Awareness & Practices	Gardening Season						Totals (n = 4901)
	
	2003 (n = 608)	2004 (n = 609)	2005 (n = 1440)	2006 (n = 777)	2007 (n = 620)	2008 (n = 847)	
Aware of pesticide bylaw*†(wgtd counts, wgtd %)	NA	NA	(n = 1452)	(n = 794)	(n = 614)	(n = 825)	(n = 3684)
Yes			734 (50.6%)	520 (65.4%)	415 (67.8%)	570 (69.2%)	2239 (60.8%)
No			133 (9.2%)	80 (10.1%)	48 (7.8%)	75 (9.1%)	335 (9.1%)
Missing, Don't Know, Refused			585 (40.3%)	195 (24.5%)	151 (24.6%)	179 (21.8%)	1110 (30.1%)

† Question pestby_1 from Pesticide Awareness Module in RRFSS. Some communities have bylaws that limit the outdoor use of pesticides, some are thinking about it and others do not. Do you think that "Name of Health Unit inserted here" currently has a bylaw that limits the outdoor use of pesticides?

**Table 5:** Respondent* & household^ lawn care awareness and practices, City of Toronto, 2003-2009 Only households with lawns (n, %, 95% CI for key practices).

Lawn Care Awareness & Practices	Gardening Season						Totals (n = 2726)
	
	2003 (n = 333)	2004 (n = 289)	2005 (n = 896)	2006 (n = 479)	2007 (n = 308)	2008 (n = 421)	
Lawn care company applied pesticides^ †							
Yes	49 (14.7%, 10.9 - 18.5)	40 (13.8%)	69 (7.7%)	18 (3.8%)	9 (2.9%)	19 (4.5%, 2.5 - 6.5)	204 (7.5%)
No	18 (5.4%)	27 (9.3%)	86 (9.6%)	69 (14.4%)	52 (16.9%)	66 (15.7%)	318 (11.7%)
Missing & Not Applicable	266 (79.9%)	222 (76.8%)	741 (82.7%)	392 (81.8%)	247 (80.2%)	336 (79.8%)	2204 (80.9%)

Household member applied pesticides^‡							
Yes	82 (24.6%, 20.0 - 29.3)	58 (20.1%)	145 (16.2%)	83 (17.3%)	43 (14.0%)	47 (11.2%, 8.1 - 14.2)	458 (16.8%)
No	229 (68.8%)	205 (70.9%)	708 (79.0%)	375 (78.3%)	248 (80.5%)	350 (83.1%)	2115 (77.6%)
Missing & Not Applicable	22 (6.6%)	26 (9.0%)	43 (4.8%)	21 (4.4%)	17 (5.5%)	24 (5.7%)	153 (5.6%)

Aware of Natural Lawn Care Campaign*§(wgtd counts, wgtd%)	NA	NA	(n = 977)	(n = 531)	(n = 344)	(n = 456)	(n = 2308)
Yes			359 (36.8%)	210 (39.4%)	133 (38.8%)	172 (37.8%)	875 (37.9%)
No			473 (48.4%)	300 (56.5%)	197 (57.3%)	269 (59.1%)	1239 (53.7%)
Missing, Don't Know, Refused			145 (14.9%)	22 (4.1%)	14 (3.9%)	14 (3.1%)	195 (8.4%)

Lawn care company used natural lawn care methods^°							
Yes	16 (4.8%, 2.5 - 7.1)	29 (10.0%)	67 (7.5%)	49 (10.2%)	32 (10.4%)	50 (11.9%, 8.8 - 15.0)	243 (8.9%)
No	23 (6.9%)	21 (7.3%)	46 (5.1%)	19 (4.0%)	13 (4.2%)	17 (4.0%)	139 (5.1%)
Missing & Not Applicable	294 (88.3%)	239 (82.7%)	783 (87.4%)	411 (85.8%)	263 (85.4%)	354 (84.1%)	2344 (86.0%)

Household member used natural lawn care methods^¶	NA						
Yes		131 (45.3%, 39.6 - 51.1)	518 (57.8%)	290 (60.5%)	196 (63.6%)	279 (66.3%, 61.7 - 70.8)	1414 (51.9%)
No		130 (45.0%)	333 (37.2%)	163 (34.0%)	97 (31.5%)	109 (25.9%)	832 (30.5%)
Missing & Not Applicable		28 (9.7%)	45 (5.0%)	26 (5.4%)	15 (4.9%)	33 (7.8%)	480 (17.6%)

† Question pbl_3 from Pesticides and Lawns Module in RRFSS. Did the lawn care company use any pesticides on your lawn to kill weeds or insects?

‡ Question pbl_8 from Pesticides and Lawns Module in RRFSS. There are also many commercial pesticides available off the shelf, such as Roundup, Killex and Weed and Feed, for HOME AND GARDEN use. Now some questions about these types of pesticides.

If pbl_2 = 1 (if hired/paid a lawn care company)Besides the services provided by the lawn care company, so far this year*, have you or someone else in YOUR HOUSEHOLD used pesticides on your LAWN to get rid of weeds or insects?

If pbl_2 = 5 (if not hired/paid a lawn care company]So far this year*, have YOU or someone else in YOUR HOUSEHOLD used pesticides on your LAWN to get rid of weeds or insects?

§ Question ng_1 from Pesticide Campaigns Module in RRFSS. Have you seen or heard ANYTHING about the Naturally Green Campaign in your community? The campaign includes lawn signs, brochures, and ads on the radio which encourage people to avoid pesticides and try pesticide free methods.

° Question pbl_7 from Pesticides and Lawns Module in RRFSS. [PBL_6 = No]Did they (the lawn care company) use any natural lawn care/pesticide-free methods?/[PBL_6 = Yes] And did they use it?

¶ Question pbl_10 from Pesticides and Lawns Module in RRFSS.

If pbl_2 = 1 (if hired/paid a lawn care company) Besides the services provided by the lawn care company, have YOU or someone else in YOUR household used pesticide-free methods such as hand weeding or used products such as corn gluten on your lawn?

If pbl_2 = 5 (If not hired/paid a lawn care company), So far this year*, have YOU or someone else in YOUR household used pesticide-free methods such as hand weeding or used products such as corn gluten on your lawn?

Among households that indicated that they had reduced their pesticide use (data not in tables), the primary reason given was for health or environmental reasons (33.7% average), followed closely by the pesticide bylaw (23.9% average) and that simply their lawn did not require pesticides (16.3% average) [53]. While the pesticide bylaw was not the most influential factor, an upward trend (+5.1%) of citing the bylaw as the motivation was seen between gardening seasons 2006 and 2007. Among households that indicated they had changed towards increasing their use of natural lawn care methods, the primary influence was health or environmental reasons (42% average). The pesticide bylaw was cited as the reason by 20% of respondents. Among those never having used natural lawn care methods, the largest group (48.3%) reported not having much knowledge of natural lawn care practices or methods.

In bivariate analyses (Table 6), awareness of the pesticide bylaw and the Natural Lawn Care Campaign were moderately associated (Odds Ratio (OR) > 2 most seasons) so either one or the other had to be used in multivariable models. Dependent variables showed variation by respondent gender and education, and household income and location, so were included in multivariable logistic models (table 6). Male respondents were generally more aware of the pesticide bylaw (OR 1.2) and less aware of the Natural Lawn

**Table 6:** Logistic regression models of variables associated with respondent awareness or household practice outcomes, weighted with individual level variable weight (Odds Ratio, [Standard Error], significant coefficients bolded).

Independent variables	Dependent variable					
	
	Respondent aware of pesticide bylaw (n = 1804)	Lawn care company applied pesticides (n = 375)	Household member applied pesticides (n = 1863)	Respondent aware of Natural Lawn Care Campaign (n = 1804)	Lawn care company used natural lawn care methods (n = 196)	Household member used natural lawn care methods (n = 1283)
**Gender **(Woman referent)						
Man	**1.21 [0.17]**	**1.67 [0.25]**	**1.71 [0.12]**	**0.85 [0.11]**	0.77 [0.37]	**0.75 [0.12]**

**Education **(completed post-2ndy referent)						
< high school	**1.52**	0.75 [0.53]	**0.72 [0.27]**	1.02 [0.24]	0.34 [1.06]	**0.52 [0.27]**
highHigh school	**[0.39]**	0.98 [0.35]	**0.77 [0.17]**	**0.74 [0.15]**	1.16 [0.48]	0.89 [0.16]
> high school	1.01 [0.23]	**1.52 [0.46]**	1.04 [0.22]	0.94 [0.20]	0.38 [0.73]	0.97 [0.24]
	1.03 [0.29]					

**Household income **(≥ LICO referent)						
< low income cutoff	1.12 [0.24]	**0.59 [0.38]**	0.80 [0.20]	**0.72 [0.16]**	**0.26 [0.62]**	**0.51 [0.19]**

**Municipality **(East York referent)						
Etobicoke	0.65 [0.36]	1.30 [0.60]	**1.37 [0.30]**	**1.31 [0.23]**	1.07 [0.79]	1.15 [0.28]
North York	**0.60**	**1.65 [0.58]**	**1.85 [0.28]**	0.94 [0.22]	1.67 [0.76]	**0.60 [0.26]**
Old City of Toronto	**[0.34]**	0.79 [0.62]	0.94 [0.29]	1.14 [0.21]	**2.26 [0.80]**	1.12 [0.26]
Scarborough	0.78 [0.34]	**2.48 [0.58]**	1.92 [0.28]	0.94 [0.22]	**6.23 [0.78]**	0.90 [0.26]
York	1.15[0.36]	1.17 [1.32]	1.15 [0.38]	**1.75 [0.30]**	1.12 [1.25]	1.20 [0.36]
Other	**0.42 [0.45]** 0.18 [1.40]	NU	3.51e-05 [393.7]	1.02 [1.35]	NU	NU

**Gardening period**(referent year)	(2005)	(2003)	(2003)	(2005)	(2005)	(2005)
2004	NA	**0.49 [0.44]**	0.97 [0.23]	NA	NA	NA
2005	NA	**0.21 [0.42]**	**0.51 [0.21]**	NA	NA	NA
2005 Oversample	1.13 [0.29]	**0.43 [0.46]**	0.81 [0.22]	**0.77 [0.20]**	**1.60 [0.52]**	**1.41 [0.19]**
2006	1.08 [0.24]	**0.08 [0.53]**	**0.59 [0.24]**	1.04 [0.16]	**3.99 [0.62]**	**1.30 [0.19]**
2006 Oversample	**1.81 [0.48]**	0.02 [1.09]	0.71 [0.29]	**0.52 [0.28]**	1.44 [0.70]	**2.29 [0.26]**
2007	**1.53 [0.27]**	**0.06 [0.53]**	**0.53 [0.24]**	**0.81 [0.16]**	**2.31 [0.56]**	**2.22 [0.20]**
2008	**1.40 [0.23]**	**0.10 [0.46]**	**0.47 [0.23]**	**0.68 [0.15]**	**5.18 [0.58]**	**2.01 [0.19]**

**Others**						
Pesticide bylaw awareness	NU	NU	NU	**2.53 [0.18]**	NU	NU
Natural Lawn Care Campaign lawn care campaign awareness	**2.55 [0.18]**	NU	NU	NU	**1.66 [0.36]**	**1.79 [0.13]**

NA indicates not available in gardening season

NU indicates variable not used for the model because not appropriate as variable to be dependent variable, not included as wanted to include all seasons, or co-variation required selection between two awareness variable.

**Figure 3:**
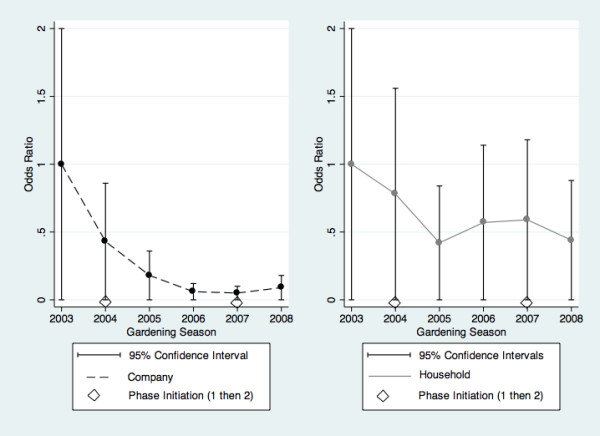
Adjusted* odds ratios (95% confidence intervals (CI)) for reported *application of pesticides *by lawn care company or by household member 2003-2008, without oversamples^. *Adjusted for respondent gender & education, household income & location. ^ Hence OR are slightly different from those in table 4

Campaign (OR 0.8). Though less likely to use natural lawn care methods, their households were more likely to apply pesticides. In contrast, among respondents with less than high school education who were also more aware of the pesticide bylaw, their households less commonly applied pesticides or used natural lawn care methods more often. Low income households were less aware of the Natural Lawn Care Campaign and applied these methods less commonly. Across gardening seasons, trends towards greater awareness of the pesticide bylaw and less application of pesticides remained, along with greater use of natural lawn care methods (but not awareness). The same pesticide application trends can be observed graphically (Figure 3), among the regular sample (without oversample, hence slightly different OR from table).

## Discussion

### Municipal Implementation

We have described an innovative approach to designing and implementing a new policy involving regulation of outdoor use of pesticides for non-essential purposes. The policy gestation over several years, the substantial efforts to engage in dialogue and debate with the public and relevant stakeholders, the mobilization of adequate TPH staff resources and the widespread dissemination of information, both about the bylaw and alternatives, reached Toronto residents. By 2008, over two-thirds of respondents reported being aware of the bylaw.

On the enforcement front, residents were active in lodging complaints, but few actual prosecutions were needed. The specialized training for health inspectors and strong enforcement presence increased awareness and likely compliance. At the same time, the complexity of governance around pesticide use, with responsibilities at the national (registration), provincial (classification as to uses and sale) and municipal levels, left an opening for the unsuccessful court challenge and created additional user confusion.

Overall costs for city taxpayers were reasonable: in the most intense launch year about 450K/2.5 million residents or CDN$0.20 per resident per year, within the lower range of the expenditure ratios reported in the review by the Canadian Centre for Pollution Prevention [21]. These were substantially lower than environmental tobacco smoke bylaw implementation and enforcement costs, where persistent conflict was greater [55,56].

### Resident practice changes

In a city where households already appeared to use pesticides less than other jurisdictions (only 15% company and 25% household application versus over 50% of households applying pesticides in other Canadian jurisdictions (Ipsos Reid, 2001, unpublished), significant further reductions in pesticide application were achieved. Comparing these falls in use is difficult given the different metrics used in the limited grey literature e.g. the drop in household contracted company applications can be framed as a modest absolute difference of 10% (14.7-4.5) or as a very large proportionate drop of 69% (10.2/14.7). Similarly for householder application, an absolute difference of 13% (24.6-11.2) or a proportionate decrease of 54% (13.4/24.6). The proportionate decreases would be comparable to the largest changes observed in the Canadian Centre for Pollution Prevention's review [21] including two other municipalities with bylaws - the very small community of Hudson, Quebec and the city of Halifax, Nova Scotia. Note that context can also influence such reductions, as comfort with pesticide use differed between rural and urban areas of Utah [57]. The absolute differences we observed would be more comparable to those achieved in Seattle and Chesapeake Bay through education and outreach alone.

However framed, we may ask why reported use did not approach zero, despite use of the suggested multiple channels in risk communication [58]. First, the bylaw permitted uses of some pesticides, which may be among those reported. Second, the continued availability of non-permitted products at stores may have led many homeowners to believe they were allowed to use them. Licensing of products is a federal mandate and the actual sales process a provincial one. Third, associations of the "perfect lawn" in suburban areas with higher socioeconomic status [14], the more status-conscious nature of a large urban centre, and the link to "men's" work in outdoor yard care are all strong North American cultural characteristics. Finally, Toronto's multi-cultural nature may have made it harder to reach the wide variety of communities who have different uses of outdoor space and whose perceptions of pesticides are influenced more by cultural practices than external information [54].

Increases in use of natural lawn care methods were not universal. This may be because alternatives to pesticides require different approaches and may not be as immediately effective. As in much health promotion, uptake of positive behaviours can be easier than relinquishing of negative ones. Corresponding changes among hired lawn care companies were modest (7.1%) perhaps attributable to the substantial investment such companies have in existing technology, and the difficulties in switching to different suppliers and techniques.

### Challenges in Evaluation

Systematic evaluation of the effectiveness of pesticide use reduction efforts including bylaws poses particularly prominent challenges. Feasible, external, independent indicators for measuring changes in pesticide use and contamination over time are limited. For example, pesticide sales (and use) data remain unavailable especially at a municipal level, except within companies. Environmental testing was conducted prior to bylaw implementation but not funded long-term. It was later funded by the provincial government (to successfully assess effectiveness of its own province-wide ban) [59].

We relied primarily on self-reported householder data on practices. Illegal activities, including pesticide use restricted under a bylaw, are generally under-reported on surveys [49]. However, householder data for all but the 2008 gardening season were collected prior to the time when residential users were subject to penalties under the bylaw, and much of the observed change occurred prior to that season. Desirability bias may also lead to over-estimation of changes in actual practices. Although this bias may have occurred with the shift in public perceptions towards use of pesticides on lawns being more socially inappropriate, such a shift would itself be a positive consequence of education and outreach efforts. There was also potential for recall bias, given that during the late fall and winter months respondents were being asked to report on practices that occurred a number of months previously. Being infrequent, pesticide applications may be more salient than other outdoor tasks, so some misclassification may be expected. However, this should be mitigated by the fact that such misclassification would not be differential across gardening seasons, as the same recall challenge would have occurred in 2003 as 2008. Other factors may reduce under-reporting, including the fact that the questions were asked as part of a longer survey with questions about many health-related topics, and that the surveys are anonymous and conducted over the telephone with an independent survey organization. On the other hand, that fact that some households reported pesticide use by companies they hired in later years indicates a risk of "over-reporting" as householders may not have been clear on permitted products or what was used.

Sampling via random digit dialling is increasingly posing challenges to representativeness with the increased use of cell phones. Further, telephone surveys face recruitment challenges as telephone advertising increases. Those achieved by RRFSS (> 50%) are on the high end, partly due to the extensive call-back procedures. Finally, conducting surveys only in English in a multi-cultural city may cause difficulties for less acculturated newcomers, though the costs of including multiple language interviewers in such surveys would be substantial and earlier ISR/RRFSS methodological work had found that the number of potential respondents 'non-functional' in English was much lower that the proportion for whom it was not their first language.

We were able to use a referent or comparison municipality in an interim examination of evaluation results for a report to City Council, showing less change in reported pesticide use observed in the community with no bylaw and smaller investment in public education [60]. However, the Toronto policy development process was extremely high profile, with widespread media attention throughout Ontario, making it highly unlikely that another community, its environmental groups and the public would be without influence from the Toronto experience. Further, the comparison municipality did not continue repeat surveys to provide data across all years as would be needed for a more rigorous comparison.

Despite these potential caveats associated with our findings, the existence of repeated measures data prior to, during and post- bylaw and education implementation is a real strength. Further, corroboration by TPH staff observations during engagements with lawn care companies, store owners and community groups, that the intent and messages of the bylaw and education program were being understood and that stakeholders were actively seeking information on pest and weed control with methods other than pesticides, assists interpretation of the repeat survey data findings. We can understand this as an *adequacy *evaluation, one that primarily seeks to assess coverage [61]. As in many environmental health interventions this one has face validity i.e. that less pesticide application will likely result in less environmental contamination and human pesticide exposure. Hence the assessment could be focused on program implementation and reductions in reported use, in a way that was highly relevant to the stakeholders involved, particularly the political representatives on the BOH and City Council [62].

### Directions

Other Canadian municipalities have followed the lead of Hudson, Halifax and Toronto; by February 2010, an estimated 154 municipalities in seven provinces had pesticide bylaws [63]. Municipal experiences with bylaw implementation were also important drivers for provincial legislation, prompting bans in Quebec, Ontario, Prince Edward Island, Nova Scotia and New Brunswick. The Toronto bylaw paved the way for broad acceptance by the public of stronger pesticide control legislation in Ontario even if it meant that pesticide products were no longer available for their personal use. It served to influence the "next step" in the evolution of public thinking about the use of pesticides. As part of a wider effort to reduce use of hazardous substances in the province, the Ontario-wide cosmetic pesticide ban, enacted on April 22, 2009, was more comprehensive in scope. It banned the sale of many common pesticides, limiting current exemptions to pesticide use, tightly restricting remaining uses and imposing larger fines and penalties, including imprisonment [64]. Building on the long policy development and implementation work of Toronto, Ontario's was an efficient regulatory process, one that other states, provinces or countries could emulate [65]. Many US states, however, have responded to the jurisdictional complexity with "pesticide pre-emption laws" [66] thereby removing the right of municipalities to pass ordinances on pesticide use.

## Conclusions

As part of environmental policy implementation, we would encourage parallel efforts to evaluate impacts. In keeping with the growing emphasis on effectiveness [67] and public accountability [68], funding should be included for indicators in different relevant domains. Comparable reporting of both absolute as well as relative changes and controlling for relevant covariates would also be helpful. The same way that toxic substance release inventories in the US [69] and in Canada [70] have paved the way for our understanding of waste releases, we would urge the development of pesticides sales databases, as a key ingredient in tracking intentional chemical inputs to humans and our ecosystems. Coupled with implementation of better exposure incident information systems [46], they should facilitate more explicit evaluations of the impacts of environmental policies and programs.

## List of Abbreviations

BOH: (Toronto) Board of Health; LICO: Low Income Cut-Off; MOH: Medical Officer of Health (for Toronto); OR: Odds Ratio; RRFSS: Rapid Risk Factor Surveillance System; TPH: Toronto Public Health.

## Competing interests

Several authors are either current (LV, RW, CM, MC) or former (MB) employees of Toronto Public Health. SW, JL and DC each had short term contracts for their work on this project with TPH. We certify that our freedom to design, conduct, interpret and publish the research is not compromised by this relationship or the funding noted below.

## Authors' contributions

MC, LV, RW, CM, DC and MB participated in conception, design, data collection, analysis, interpretation, writing, and revisions. JL and SW participated in analysis, interpretation, writing and revisions. All authors have read and critically reviewed the manuscript, accept responsibility for its contents, and agree that the final paper is ready for submission. They have given permission to DC to submit on behalf of the authors.
